# A central role for glial CCR5 in directing the neuropathological interactions of HIV-1 Tat and opiates

**DOI:** 10.1186/s12974-018-1320-4

**Published:** 2018-10-10

**Authors:** Sarah Kim, Yun Kyung Hahn, Elizabeth M Podhaizer, Virginia D McLane, Shiping Zou, Kurt F Hauser, Pamela E Knapp

**Affiliations:** 1 Department of Anatomy and Neurobiology, Virginia Commonwealth University School of Medicine, 1217 E. Marshall St, Richmond, VA 23298-0709 USA; 2 Department of Pharmacology and Toxicology, Virginia Commonwealth University, Richmond, VA 23298 USA; 3 Institute for Drug and Alcohol Studies, Virginia Commonwealth University, Richmond, VA 23298 USA; 4 Present Address: BioLegend, Inc., 210 Rustcraft Rd., Dedham, MA 02026 USA

**Keywords:** Human immunodeficiency virus, Morphine, C-C chemokine receptor 5, Maraviroc, Brain-derived neurotrophic factor, NeuroHIV

## Abstract

**Background:**

The collective cognitive and motor deficits known as HIV-associated neurocognitive disorders (HAND) remain high even among HIV+ individuals whose antiretroviral therapy is optimized. HAND is worsened in the context of opiate abuse. The mechanism of exacerbation remains unclear but likely involves chronic immune activation of glial cells resulting from persistent, low-level exposure to the virus and viral proteins. We tested whether signaling through C-C chemokine receptor type 5 (CCR5) contributes to neurotoxic interactions between HIV-1 transactivator of transcription (Tat) and opiates and explored potential mechanisms.

**Methods:**

Neuronal survival was tracked in neuronal and glial co-cultures over 72 h of treatment with HIV-1 Tat ± morphine using cells from CCR5-deficient and wild-type mice exposed to the CCR5 antagonist maraviroc or exogenously-added BDNF (analyzed by repeated measures ANOVA). Intracellular calcium changes in response to Tat ± morphine ± maraviroc were assessed by ratiometric Fura-2 imaging (analyzed by repeated measures ANOVA). Release of brain-derived neurotrophic factor (BDNF) and its precursor proBDNF from CCR5-deficient and wild-type glia was measured by ELISA (analyzed by two-way ANOVA). Levels of CCR5 and μ-opioid receptor (MOR) were measured by immunoblotting (analyzed by Student’s *t* test).

**Results:**

HIV-1 Tat induces neurotoxicity, which is greatly exacerbated by morphine in wild-type cultures expressing CCR5. Loss of CCR5 from glia (but not neurons) eliminated neurotoxicity due to Tat and morphine interactions. Unexpectedly, when CCR5 was lost from glia, morphine appeared to entirely protect neurons from Tat-induced toxicity. Maraviroc pre-treatment similarly eliminated neurotoxicity and attenuated neuronal increases in [Ca^2+^]_i_ caused by Tat ± morphine. proBDNF/BDNF ratios were increased in conditioned media from Tat ± morphine-treated wild-type glia compared to CCR5-deficient glia. Exogenous BDNF treatments mimicked the pro-survival effect of glial CCR5 deficiency against Tat ± morphine.

**Conclusions:**

Our results suggest a critical role for glial CCR5 in mediating neurotoxic effects of HIV-1 Tat and morphine interactions on neurons. A shift in the proBDNF/BDNF ratio that favors neurotrophic support may occur when glial CCR5 signaling is blocked. Some neuroprotection occurred only in the presence of morphine, suggesting that loss of CCR5 may fundamentally change signaling through the MOR in glia.

## Background

Human immunodeficiency virus type 1 (HIV-1) remains a global epidemic [1]. Despite significant antiretroviral suppression of HIV-1 propagation in the periphery, limited penetration of combination antiretroviral therapy (cART) drugs through the blood-brain barrier [2, 3] as well as the early viral integration into the host genome cultivates a reservoir in which low levels of viral replication can be sustained in the central nervous system (CNS) [4–6]. Thus, HIV-1+ individuals are especially vulnerable to CNS injury, which afflicts as many as 50% of this population [7–9]. The neurological consequences of HIV-1 infection are known collectively as HIV-associated neurocognitive disorders (HAND). HAND presents as a spectrum of deficits ranging from mild or asymptomatic cognitive disorders to severe, HIV-associated dementia and includes a variety of cognitive, behavioral, and/or motor symptoms [10]. Postmortem findings in HIV-infected individuals, even those effectively treated with cART, often include signs of prominent CNS inflammation, such as increased numbers and/or activation of microglia and astroglia, perivascular inflammation, and leukocytic infiltration resulting in marked neuronal degeneration [11–13]. Notably, neuronal injury and alterations in signaling are not accompanied by direct viral infection of neurons [14–16]. Instead, microglia and macrophages are the major source of productive viral infection in the CNS [17–19]. Small numbers of astrocytes are infected and can produce toxic proteins that injure bystander neurons, but they have not been reliably shown to produce virus [20, 21]. These combined findings highlight the importance of glial impact on neurons, which is normally critical in maintaining proper neuronal activity and survival and suggests a mode of indirect injury that is a consequence of the innate CNS immune response to HIV.

HIV-1 infection and injection drug use are interlinked epidemics, due in large part to needle sharing and increased risky sexual behavior. Because heroin (diacetyl morphine) is widely abused and its active metabolite, morphine, is an opiate prescribed for pain syndromes experienced by HIV patients, we and others are interested in neurological interactions of HIV-1 and heroin/morphine. The comorbid effects of HIV and opiates are not trivial. HIV+ individuals who also abuse opiates demonstrate more severe neuropathology than those who do not, and these findings can translate to exacerbated and accelerated HAND [22–25]. The actions of morphine in this context occur primarily through the activation of μ-opioid receptors (MORs) expressed on glial cells. MOR activation results in the potentiation of HIV-induced release of pro-inflammatory factors (e.g., TNFα, IL-1β, IL-6, CCL5, and CCL2), as well as oxidative and nitrosative stress, mitochondrial dysregulation, elevated intracellular calcium levels, and excess extracellular glutamate (via restriction of astroglial glutamate uptake), all of which promote neurotoxicity [26–30]. Dysregulated release of inflammatory factors upon chronic exposure to viral proteins may recruit and activate more immune cells, propagating a cycle of increasing inflammation with significant downstream neuronal consequences.

One receptor vulnerable to the aforementioned dysregulation by HIV infection is C-C chemokine receptor 5 (CCR5). CCR5 is widely expressed on T lymphocytes, macrophages, microglia, and dendritic cells and plays a critical role in inducing migration of immune cells to sites of infection and injury in response to elevated levels of certain C-C-chemokine ligands (MIP-1α/CCL3, MIP-1β/CCL4, CCL5/RANTES) [31]. CCR5 and its ligands are upregulated during HIV infection, leading to excess activation of CCR5-expressing cells, including CNS microglia and astrocytes [32, 33]. A homozygous deletion of 32 base pairs in the CCR5 gene prevents its expression on the cell surface and confers improved but not absolute immunity to infection with R5-tropic strains of HIV-1 [34]. Individuals carrying the allele show slowed disease progression upon infection with HIV and less cognitive impairment [35–38]. Furthermore, maraviroc, a CCR5 antagonist with relatively high CNS penetrance [39], reduces microglial activation in the simian immunodeficiency virus-infected model to uninfected control levels and reduces the expression of several pro-inflammatory factors [40]. cART regimens that are supplemented with maraviroc improve the neurocognitive status of HIV+ patients and reduce CSF levels of TNFα [41, 42]. Morphine can alter CCR5 expression by monocytes and activated T cells, contributing to increased viral entry and replication, and excess CNS immune activation [43, 44]. We previously demonstrated that loss of CCL5, a CCR5 ligand, prevented widespread glial activation and reduced levels of another inflammatory ligand (CCL2), suggesting CCL5 may be an upstream activating signal that promotes the expansion of downstream pro-inflammatory responses [45]. The present studies investigate whether interrupting CCR5 signaling may protect neurons against the comorbid effects of HIV-1 and opiate exposure, apart from any effect of blocking HIV entry. We demonstrate using mixed glial-neuronal co-cultures that morphine potentiates Tat-induced neuronal death and that a loss of CCR5 expression on glial cells rescues neurons from such enhanced neurotoxicity. Surprisingly, morphine completely protected against Tat neurotoxicity in cultures with CCR5-null glia even though Tat by itself was still toxic. Levels of brain-derived neurotrophic factor (BDNF) are reduced in HIV+ individuals and by glycoprotein 120 (gp120), an HIV-1 envelope protein with neurotoxic properties [46, 47]. We found that the ratio of neurotrophic BDNF to its neurotoxic precursor (proBDNF) was altered in CCR5-null glia exposed to Tat and morphine co-treatment such that the environment favored neuronal support. BDNF also rescued neurons from Tat + morphine neurotoxicity in a manner similar to the loss of glial CCR5 expression. Overall, we postulate that CCL5/CCR5 signaling is a point of convergence for opiate-Tat interactions within the inflammatory milieu of the HIV-infected CNS. Blocking CCR5 appears to enhance neuroprotection, perhaps by increasing BDNF-related neuroprotection. Inactivation or loss of CCR5 may also change heterologous interactions between MOR and CCR5 related to toxicity and protection.

## Methods

Experiments were conducted in compliance with procedures reviewed and approved by the Virginia Commonwealth University Institutional Animal Care and Use Committee.

### CCR5-null mice

**Fig. 1:**
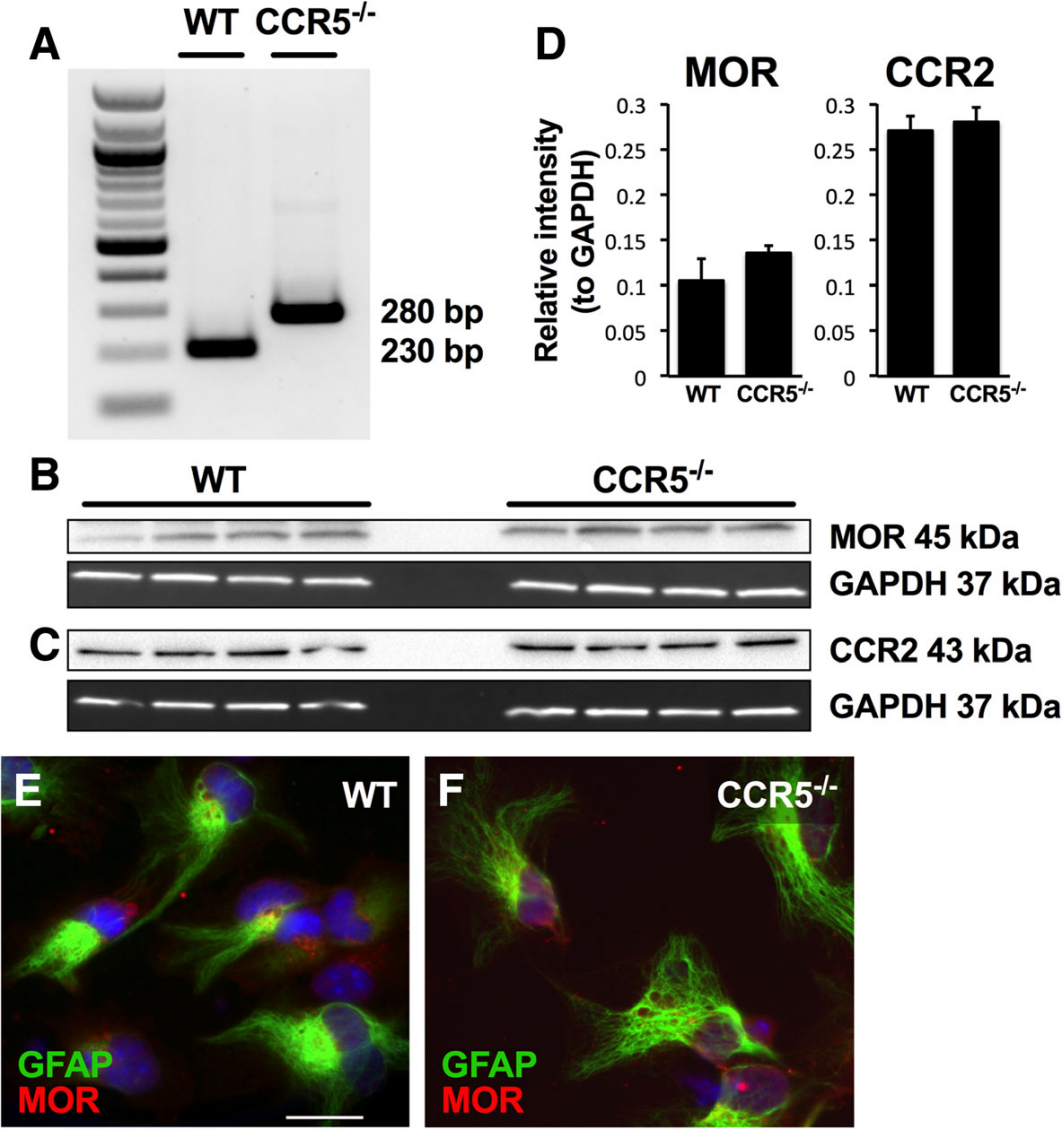
Expression of MOR and CCR2 are not altered in cell cultures derived from CCR5-null mice. **a** The CCR5 knockout was verified by the presence of a 280-bp band that represents the neomycin resistance gene insertion into the single coding exon of the receptor. A 230-bp band indicates the presence of the CCR5 receptor in wild-type mice. A 100-bp ladder is shown for reference. **b**, **c** MOR and CCR2 protein levels were assessed in both wild-type and CCR5-deficient glia and normalized against GAPDH. **d** Statistical comparison (two-tailed Student’s *t* test) demonstrated no significant difference between genotypes in levels of either receptor. **e**, **f** Immunocytochemistry of wild-type (**e**) and knockout (**f**) glia show similar expression of MOR (red) in GFAP+ (green) astrocytes. Astrocyte morphology varies widely in the cultures. MOR appears as a punctate distribution along the cell surfaces as well as in cytoplasmic areas. Scale bar = 20 μM

Transgenic mice in which there has been a loss in CCR5 expression were obtained from Jackson Labs (Bar Harbor, ME) and maintained as homozygous breeding trios. Briefly, the insertion of a neomycin resistance gene to replace the single coding exon has resulted in the constitutive loss of CCR5 expression. To confirm the loss of CCR5, tail snips and harvested glial cells were digested and DNA was isolated as per the instructions of the manufacturer (KAPA Mouse Genotyping; KAPA Biosystems; Wilmington, MA). Primer sets designed to identify the neomycin resistance gene as well as the CCR5 coding exon were obtained from Jackson Labs. Polymerase chain reaction was carried out to confirm the presence of the neomycin resistance gene and the absence of the CCR5 sequence (Fig. 1a). Because the knockout is global, CCR5-deficient glia or neurons are reconstituted into co-cultures with wild-type neurons or glia, respectively, to study effects of mutations in a single cell type. Mice of the C57Bl6/J background strain were used as wild-type controls. The CCR5-null mice displayed no overt signs of illness or problems during development, and litters occur in a similar frequency and size as the C57Bl6/J strain.

### Primary co-cultures of mixed glia and striatal neurons

Mixed glial cultures (approximately 90% astroglia, 8% microglia, and 2% glial progenitors/incipient oligodendroglia [30]) were obtained from C57Bl6/J or CCR5-deficient pups at 0–1 day postnatal. Whole brains were dissected, minced, and incubated with trypsin (2.5 mg/ml) and DNase (0.015 mg/ml) in Dulbecco’s modified Eagle’s medium (Invitrogen, Carlsbad, CA; 30 min, 37 °C). Tissue was resuspended in medium containing 10% fetal bovine serum (Hyclone, Logan, UT), triturated, and filtered twice through 100 μm and 40 μm pore nylon mesh, then plated onto poly-L-Lysine-coated (Sigma-Aldrich, St Louis, MO; 0.5 mg/ml) 24-well plates at a density of 75 × 10^3^ cell/well. Glia reached confluency after 7–8 days, after which neurons were plated onto their surface.

Neurons were cultured from striata dissected from C57Bl6/J or CCR5-deficient mice at gestational days 15–17. Tissue was incubated (30 min, 37 °C) with trypsin (2.5 mg/ml) and DNase (0.015 mg/ml) in neurobasal medium (Invitrogen), supplemented with B-27 (Invitrogen), L-glutamine (9.5 mM; Invitrogen), glutamate (25 μM; Sigma), and antibiotic/antimycotic solution containing penicillin, streptomycin, and amphotericin B (Invitrogen). After centrifugation, the tissue was triturated and filtered twice through a 70 μm pore nylon mesh to achieve a single-cell suspension. The cells were then plated on top of a bed of confluent glia at 25 × 10^3^ cells/well, and the co-cultures were grown for another 7–8 days in the supplemented neurobasal medium. At this point, the neurons were relatively mature, as previously assessed by the expression of microtubule-associated protein 2 and an array of receptors such as NMDA-R and, importantly, the opiate receptors. Mature neurons also take on a distinct morphology compared to neural progenitor cells, possessing a larger cell body with a prominent nucleus and established axonal and dendritic processes.

### Repeated measures assessment

**Fig. 2:**
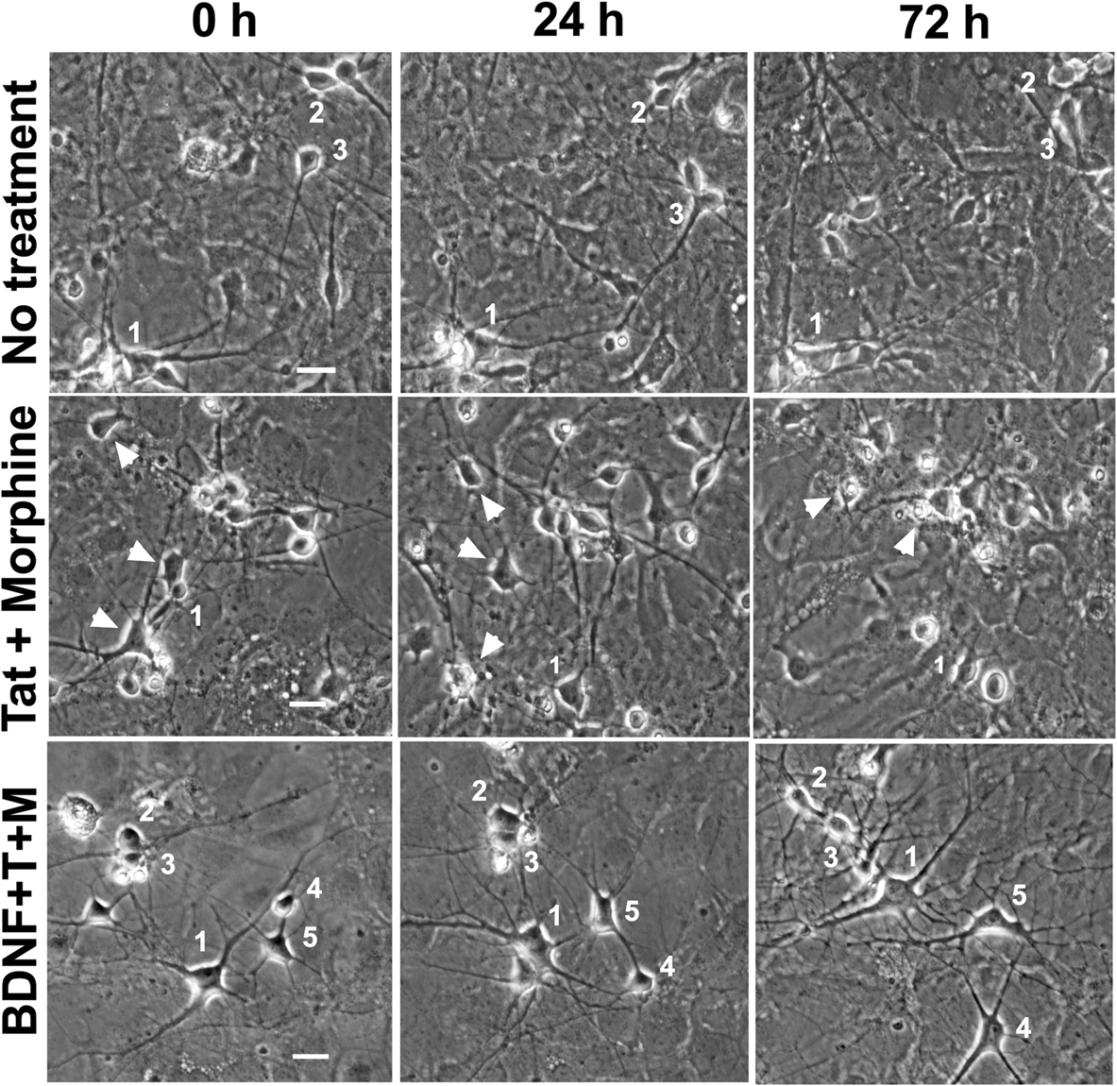
Representative time-lapse images track neuronal fate. Time-lapse images of co-cultures of wild-type neurons and glia were taken every hour to track neuron survival. The fate of individual neurons can be tracked over time by following the labeled number in the image. Neurons were pre-selected in the image taken at 0 h and followed for the duration of the experiment or until time of death. Death was determined using a set of rigorous morphological criteria including loss of phase brightness, fragmentation of neurites, and collapse of the cell soma (white arrowheads). Representative images from three treatment groups (no treatment, Tat + morphine, BDNF + Tat + morphine) are shown here. Wells receiving no treatment typically showed 85–90% survival rates. Data in Figs. 3 and 6 show that Tat + morphine treatment results in a higher frequency of neuron death by 72 h, and BDNF rescues neurons from the death induced by Tat + morphine treatment. Scale bars for each set of panels  = 20 μM

Each plate was maintained in a temperature-controlled, CO_2_-regulated chamber (37 °C, 5% CO_2_) in a heat insert MXX holder (PeCon Instruments, Erbach, Germany) and placed on the scanning stage of a Zeiss Axio Observer Z1 inverted microscope (Carl Zeiss, Inc., Thornwood, NY). Five to ten non-overlapping fields, each containing five to ten striatal neurons, were selected from each well based on distinctive neuronal morphology, including features listed in the previous section. Time-lapse images of the selected fields were recorded in 1-h intervals for 72 h using an automated, computer-controlled stage encoder and Axiovision 4.6 software (Carl Zeiss, Inc.). Pre-selected neurons were followed over the 72-h time course and assessed for survival in each hourly image. Neuronal death was determined through morphological criteria, including neurite disintegration, loss of phase brightness, and involution or complete fragmentation of the cell body, all of which were present in each cell counted for analysis (Fig. 2). The number of viable neurons was binned into 4-h intervals for analysis (see the “Statistical Methods” section).

### Intracellular calcium assessment

Co-cultures of mixed glia and neurons were prepared on 12-well glass-bottom MatTek (Ashland, MA) plates for calcium imaging. Cells were loaded with 1-μM Fura-2 AM (Invitrogen, Carlsbad, CA) suspended in DMSO for 30 min then washed. This concentration was optimized for minimal astrocyte loading to reduce background signals. The plate was incubated for 30 min in a temperature- and CO_2_-regulated chamber (37 **°**C, 5% CO_2_) to ensure de-esterification of the acetoxy methylester (AM) group. On a computer-controlled stage embedded in a Zeiss Axio Observer Z1 microscope, two 20X fields containing five to ten neurons were identified in each well and imaged using Zeiss Zen software (Carl Zeiss Microscopy, LLC, Thornwood, NY). Cells were treated with Tat ± morphine ± maraviroc, and a series of fluorescent images of the same neurons (excitation at 340 and 380 nm, emission at 510 nm) was taken every 15 min for 1 h. Two regions of interest (ROIs; each 15–20 pixels), positioned to avoid both the nucleus and margins of the cell, were selected within the scant cytoplasm of all neurons. ROIs were selected in neurons based on Fura-2 loading but prior to ratiometric imaging.

### HIV-1 Tat and drug treatments

Cultures were treated with Tat_1–86_ Clade IIIB (1.2 ng/μl; ImmunoDiagnostics, Woburn, MA), morphine sulfate (500 nM; NIDA Drug Supply System), the broad-spectrum opioid receptor antagonist naloxone (1.5 μM; Sigma), or maraviroc (50 nM; BOC Sciences, Shirley, NY), a small molecule, allosteric inhibitor of CCR5, as well as a number of drug combinations. Tat and morphine were added concurrently; naloxone and maraviroc were given 1 h prior to Tat and morphine treatments. To explore compensatory repercussions of a constitutive knockout, we compared maraviroc treatments to CCR5-deficient cultures in two different paradigms. In short-term experiments, maraviroc was added immediately prior to the start of the time-lapse imaging. In long-term experiments, maraviroc was added to the media for the duration of the co-culture maturation and imaging period (approximately 2 weeks) and refreshed every 2–3 days. The Tat concentration was chosen based on prior studies that similarly showed neuron death within the 72-h period [30]. Morphine, naloxone, and maraviroc concentrations were based on previous studies from this lab and chosen to produce full receptor occupancy or antagonism. BDNF (50 ng/ml) was purchased through Sigma-Aldrich.

### ELISA

C57 wild-type or CCR5-deficient glia were matured for 7–8 d and then treated with Tat and/or morphine for 6 or 24 h. Media was harvested and immediately stored in − 80 °C. The conditioned media were assessed for BDNF (Abcam, Cambridge, UK) and proBDNF (Biosensis, Thebarton, Australia) by ELISA according to manufacturers’ instructions. 3,3′,5,5′-tetramethylbenzidine substrate was added for color development, and plates were read at 450 nm on a SpectraMax M2 microplate reader (Molecular Devices, San Jose, CA) immediately after terminating the reaction, then analyzed using SoftPro Max 1.6 software. BDNF and proBDNF levels were determined based on a standard curve.

### Immunoblotting

CCR2 and MOR proteins were assessed in cultured wild-type C57 and CCR5-knockout glial cells. Cells were harvested in lysis buffer containing 1× Tris-buffered saline (TBS), 1% NP-40, 1% Triton X-100, 1 mM PMSF, 10% glycerol, and Halt Protease Inhibitor Cocktail (Thermo Fisher Scientific, Waltham, MA), centrifuged (15 min, 40,000 rpm) and stored at − 80 °C until use. Protein concentration of each sample was measured using the BCA protein assay (Thermo Fisher Scientific). Forty μg of protein lysates were loaded into each well of a 4–20% Tris-HCl Ready Gel (Bio-Rad Laboratories, Hercules, CA) along with Precision Plus Protein Dual Color Standards (Bio-Rad; MW range 10–250 kDa) to visualize protein transfer and determine molecular weight. Proteins were transferred to PVDF membranes (Bio-Rad). Antibodies to CCR2 (1:1000, Abcam), MOR (1:1000, Antibodies Incorporated), and GAPDH (1:2000, Abcam) were used to probe the blots. Fluorescent secondary antibodies were then visualized on a ChemiDoc Gel Imaging system and analyzed with Bio-Rad Image Lab Software 5.2.1. CCR2 and MOR values were normalized to GAPDH.

### Immunocytochemistry

Wild-type and CCR5-deficient glial cultures were grown to confluence on glass coverslips, fixed in 4% paraformaldehyde and permeabilized (0.1% Triton X-100, 1% normal goat serum) for 15 min. After blocking for 1 h (1% normal goat serum, 0.1% BSA), primary antibodies to GFAP (1:1000, Millipore) and MOR (1:500, Antibodies Incorporated, Davis, CA) were used to label astrocytes and MOR, after which nuclei were identified by the Hoechst 33342 stain (1:20,000). Coverslips were mounted on glass microscope slides with Prolong Gold anti-fade reagent (Invitrogen).

### Statistical methods

Time-lapse studies tracking neuron survival were analyzed by repeated measures ANOVA (Graph Pad Prism 7). The number of viable neurons was binned into 4-h intervals and analyzed to compare treatment effects. Bonferroni’s post hoc test was used to determine group differences following confirmation of main effects. Findings are presented as a mean percentage of viable neurons relative to the total number of pre-selected neurons. Intracellular calcium levels were calculated as a percent of control, defined as untreated neurons measured at baseline. Mean effects of Tat ± morphine ± maraviroc on [Ca^2+^]_i_ in individual neurons were analyzed at 15-min intervals up to 1 h using repeated measures ANOVA with Duncan’s post hoc testing (Statistica 13.2, Dell Inc., Tulsa, OK). ELISA results were analyzed using two-way ANOVA followed by Fisher’s PLSD post hoc testing to assess individual group differences. Protein expression levels of MOR and CCR2 were statistically compared using a two-tailed Student’s *t* test. An alpha level of *p* ≤ 0.05 was considered significant for all tests. Data are expressed as mean values ± standard error of the mean (SEM).

## Results

### Characterization of CCR5, MOR, and CCR2 expression in wild-type and CCR5-deficient cultures

DNA and protein analysis were used to characterize expression of CCR5, MOR, and CCR2 in both wild-type and CCR5-deficient mixed glial cultures. We first confirmed that cultures derived from CCR5-null transgenic mice did not express CCR5 using PCR (Fig. 1a). Western blots showed that MOR protein levels did not differ between wild-type and CCR5-null glial cultures when MOR expression was examined as a fraction of GAPDH (Fig. 1b, d). Immunocytochemical labeling of astrocytes expressing both GFAP and MOR demonstrates similar morphological distribution of MOR irrespective of CCR5 expression, namely, both cytoplasmic and more superficial punctate distribution near the cell body and along processes (Fig. 1e, f). Because of the constitutive loss of CCR5 in the transgenic mice, expression of a closely related chemokine receptor, CCR2, was examined to investigate the possibility of compensatory co-regulation. Western blot analysis showed no differences in CCR2 expression across several samples (*n* = 4) of wild-type and CCR5-null glia (Fig. 1c, d). Relative intensities for both CCR2 and MOR were quantified (Fig. 1d) and analyzed using a two-tailed Student’s *t* test.

### Enhanced HIV-1 Tat and opiate neurotoxicity reversed by loss of glial CCR5

**Fig. 3:**
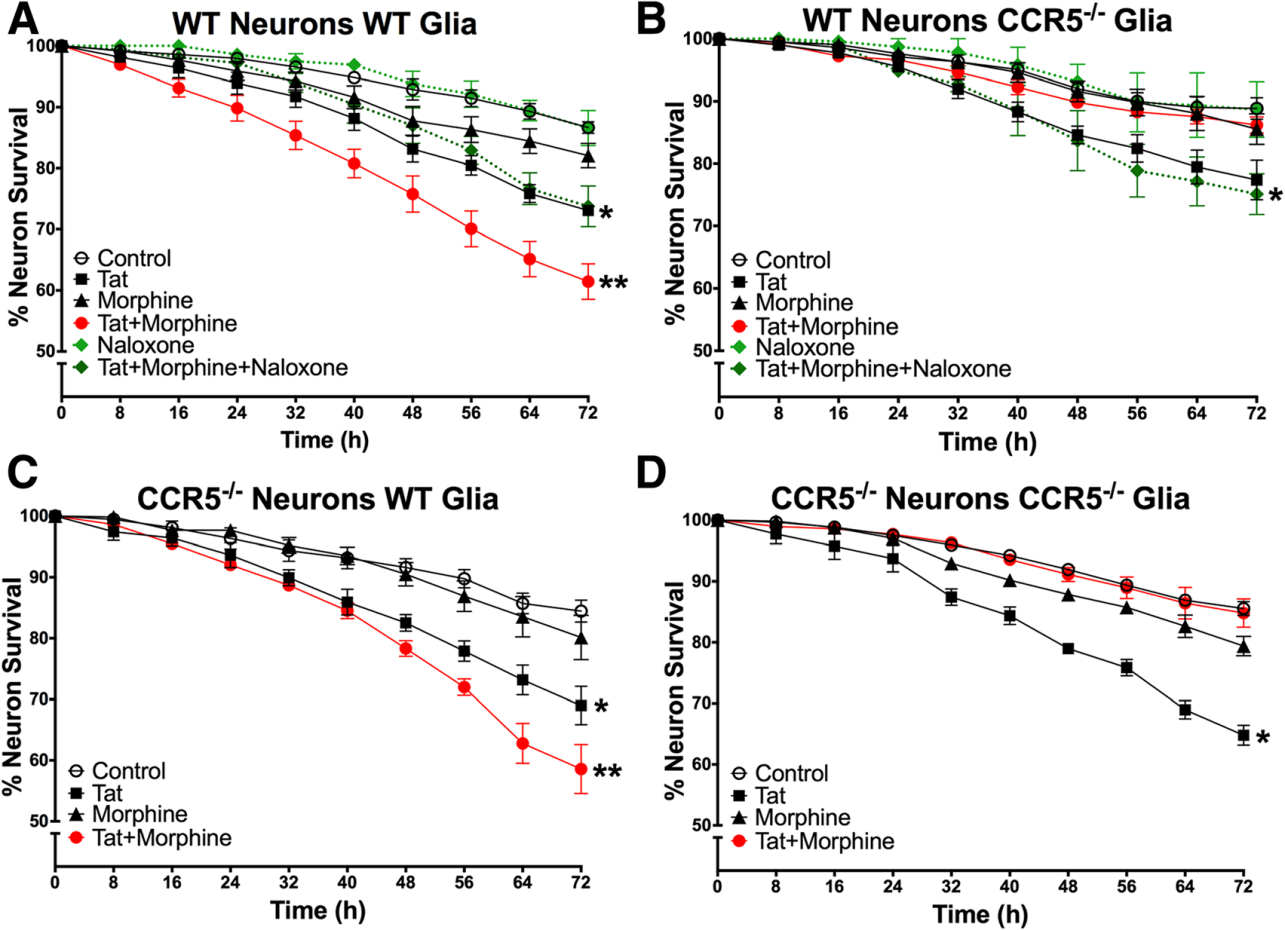
Neurotoxic effects of HIV-1 Tat and morphine are reversed by loss of glial CCR5. **a** In C57Bl6/J wild-type co-cultures, Tat is neurotoxic (*p = 0.001 vs control), and co-exposure to morphine enhanced Tat-induced toxicity over a 72-h period (**p < 0.001 vs control, *p* < 0.05 vs Tat). This interaction was blocked by pretreatment with naloxone, a broad-spectrum opioid receptor antagonist. Naloxone or morphine by themselves had no effect on neuronal survival (*n* = 4–8). **b**–**d** To explore the role of CCR5 in mediating neurotoxic interactions between Tat and morphine, co-cultures in which glia, neurons, or both were deficient in CCR5 were established. **b** In co-cultures where glia are CCR5-null but neurons are wild-type, exposure to Tat by itself still led to significant neurotoxicity (*p < 0.001 vs control); however, the morphine-enhanced neurotoxicity seen in wild-type cultures was eliminated. In fact, morphine co-treatment entirely abolished Tat toxic effects, restoring neuronal survival to control levels. Pre-treatment with naloxone re-established Tat toxicity, suggesting that actions at the μ-opioid receptor mediate this neuroprotection (*n* = 4–8). **c** In co-cultures where neurons are CCR5-null but glia are wild-type, the survival curves are similar to wild-type co-cultures (*n* = 5). **d** In co-cultures between CCR5-deficient glia and neurons, the survival curves are similar to co-cultures where only glia were CCR5-deficient (*n* = 5). Overall, the results from the CCR5-deficient co-cultures suggest an important role for glial CCR5 in the neurotoxic interactions of HIV-1 Tat and opiates that act at the MOR

In order to investigate the role of CCR5 in mediating the neurotoxic interactions between Tat and morphine, we established a series of co-cultures in which the glia, neurons, or both lacked CCR5. Representative images shown in Fig. 2 illustrate the fate of selected neurons when tracked over the 72-h trial. Using C57Bl6/J wild-type co-cultures, we confirmed that Tat is neurotoxic and that interactions with morphine enhanced Tat-induced toxicity over a 72-h period [30] (Fig. 3a). These effects appear to be mediated by opioid receptors, most likely MOR, since pre-treatment with the broad-spectrum opioid receptor antagonist naloxone eliminated Tat and morphine interactions. In cultures where glia did not express CCR5, exposure to Tat by itself still led to significant levels of neurotoxicity (Fig. 3b). However, the presence of morphine had unexpected effects. When CCR5 was absent from glia, the neurons were protected from Tat and morphine interactions as hypothesized. Surprisingly, in the presence of morphine, the neurotoxic effects of Tat were completely abolished, since neurons in the Tat and morphine co-exposure group showed no additional losses compared to controls. Naloxone pre-treatment blocked the ability of morphine to exacerbate neuronal losses following Tat exposure (compare Fig. 3a, b). Importantly, naloxone also reversed the unexpected protective effects of morphine in CCR5-deficient glial cultures co-exposed to Tat. The paradoxical effect of morphine to protect against Tat neurotoxicity CCR5-deficient conditions thus appears to be mediated through actions meditated by opioid receptors. These effects were presumed specific for glial loss of CCR5, as cultures with CCR5-deficient neurons, but with CCR5-expressing glia, exhibited levels of neuronal death similar to those seen in wild-type co-cultures exposed to Tat ± morphine (Fig. 3c). Importantly, there was no effect of morphine alone on neuronal survival in any combination of the co-cultured cells. Lastly, co-cultures in which both neurons and glia were CCR5-deficient showed survival curves similar to those in which glia alone were CCR5-deficient (Fig. 3d). Overall, these results suggest an important role for CCR5-expressing glia, as well as the importance of CCR5 and MOR interactions, in mediating the neurotoxic interactions of HIV-1 Tat and opiates.

### Constitutive CCR5 loss affects neuronal survival differently than short-term CCR5 blockade

**Fig. 4:**
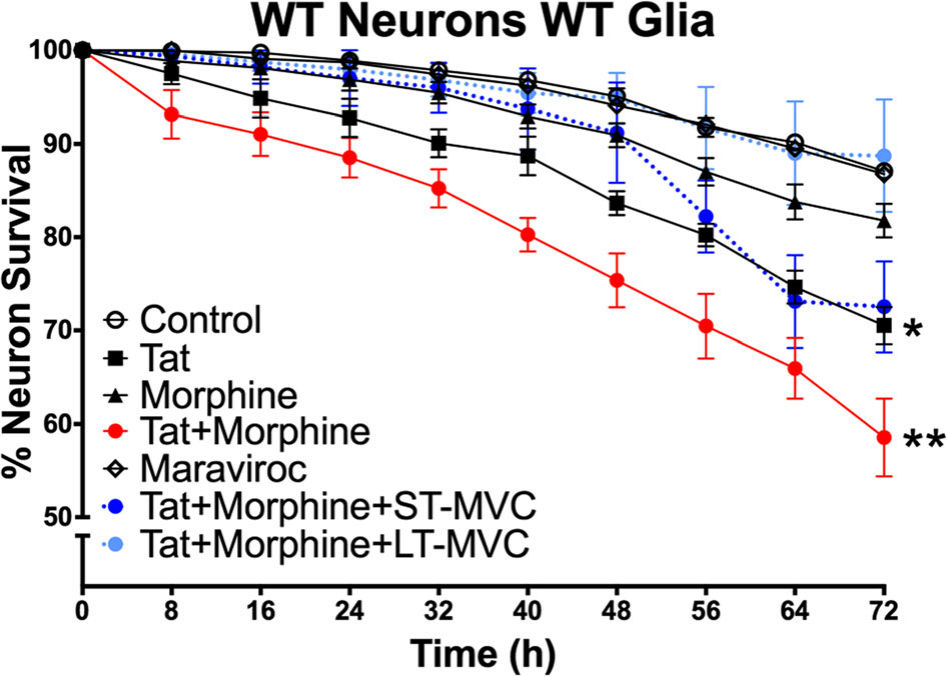
Constitutive CCR5 loss affects neuron survival differently than short-term CCR5 blockade. Maraviroc was applied to the co-culture to compare the effects of a CCR5 antagonist to a genetic knockout. Maraviroc was applied in two different paradigms that permitted us to manipulate the time period of CCR5 loss. The first was a short-term pre-treatment immediately before adding Tat and/or morphine (ST-MVC; *n* = 4); in this paradigm, maraviroc was on the cultures for a period of 72 h, during the time of Tat and morphine treatments. The second was a longer-term exposure starting 3 days after glia were plated and continuing for the entire 2-week duration of the experiment with replacement of the media every 48 h (LT-MVC; *n* = 4). The Tat + morphine + LT-MVC survival curve matched that of cultures with CCR5-deficient glia. The Tat + morphine + ST-MVC eliminated the morphine–Tat interaction and only showed a trend towards eliminating Tat toxicity (*p* = 0.08 vs control). This set of studies suggests that compensatory effects occur over time with CCR5, which dramatically alter morphine–Tat interaction and neurotoxicity

Long-term, constitutive knockout of CCR5 might result in compensatory changes during development that alter neuronal sensitivity to Tat or morphine. To explore this hypothesis, we used a paradigm where the length of CCR5 blockade was controlled using the CCR5 antagonist maraviroc (Fig. 4). Here we show that a relatively long-term, 2-week incubation with maraviroc (LT-MVC) mimicked the effects on neuron survival seen in co-cultures with CCR5-deficient glia. That is, morphine–Tat interactions that enhance neurotoxicity were negated, and morphine additionally protected completely against Tat neurotoxicity. However, shorter-term exposure to maraviroc, starting immediately before Tat and morphine were co-administered (ST-MVC), had much more limited effects. Short-term maraviroc treatment reduced the interactive effects of Tat and morphine; however, it did not reduce the neurotoxicity of Tat itself irrespective of whether morphine was present.

### Maraviroc protects against acute increases in neuronal [Ca^2+^]_i_

**Fig. 5:**
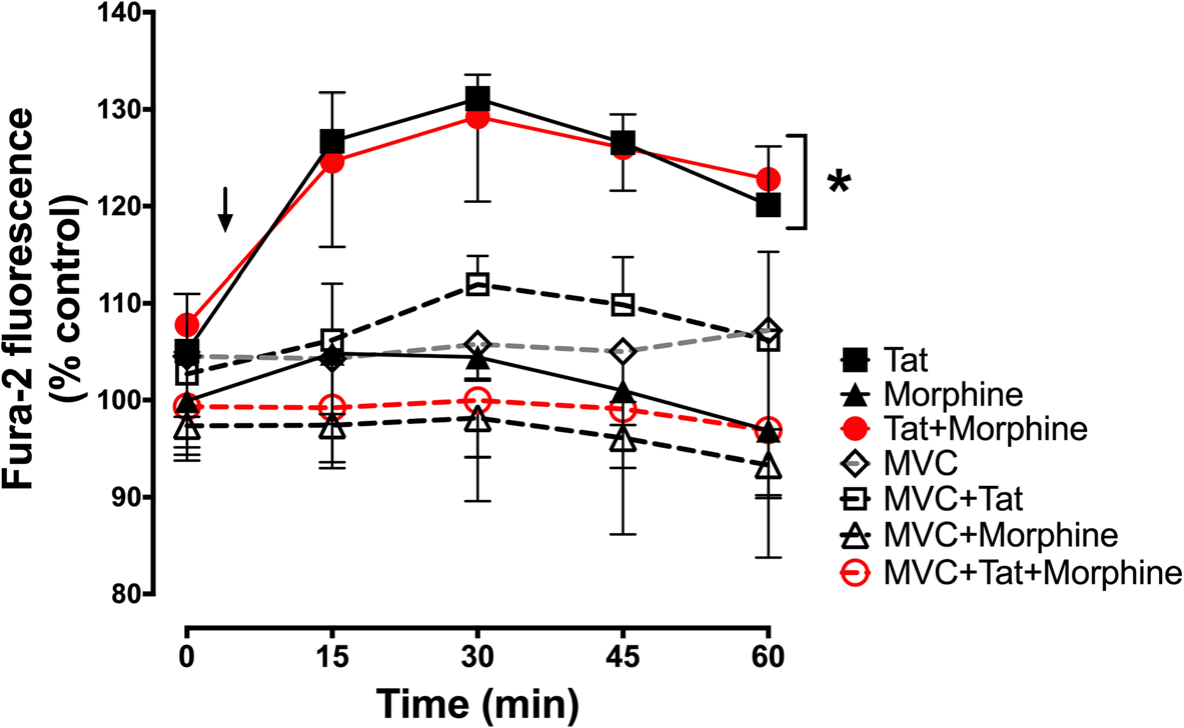
Maraviroc reduces Tat-mediated increases in [Ca^2+^]_i_. Intracellular calcium levels were assessed in neuron-glia co-cultures by ratiometric imaging of Fura-2. A series of images were taken every 15 min for 1 h to track the response of individual neurons. Initial [Ca^2+^]_i_ measurements were taken prior to any treatment at the 0-min time point. Tat and/or morphine treatments were applied 10 min prior to the second reading (marked by arrow). There were significant effects for both time (*p* = 0.001) and treatment (*p* = 0.009) when assessed by repeated measures ANOVA. Treatment with Tat or Tat + morphine (marked by asterisk) led to significant increases in [Ca^2+^]_i_, as indicated by increased F340/F380 ratios. Pre-treatment with maraviroc blocked the Tat + morphine-induced increase (*p* = 0.008; Duncan’s post hoc test) as well as the Tat-mediated response (*p* = 0.054). Morphine and maraviroc alone did not significantly alter [Ca^2+^]_i_. Results are presented as percent of the control F340/F380 ratios for each concurrent time point (*n* = 3 independent experiments)

Disruptions in calcium homeostasis are a common response to neurotoxic signals. As an indicator of how maraviroc affected neuronal function, we performed ratiometric imaging with Fura-2 to assess changes in the [Ca^2+^]_i_ level of individual neurons over a 60-min period of treatment with Tat ± morphine ± maraviroc. Tat ± morphine treatments significantly increased [Ca^2+^]_i_ by 15 min, and this was maintained for the duration of the trial (Fig. 5). Importantly, even at this early time point, co-exposure to maraviroc blocked the changes, suggesting that reduced CCR5 signaling had the effect of maintaining normal [Ca^2+^]_i_ levels and stabilizing neuronal function.

### BDNF protects against Tat toxicity and HIV-1 Tat and morphine interactions

**Fig. 6:**
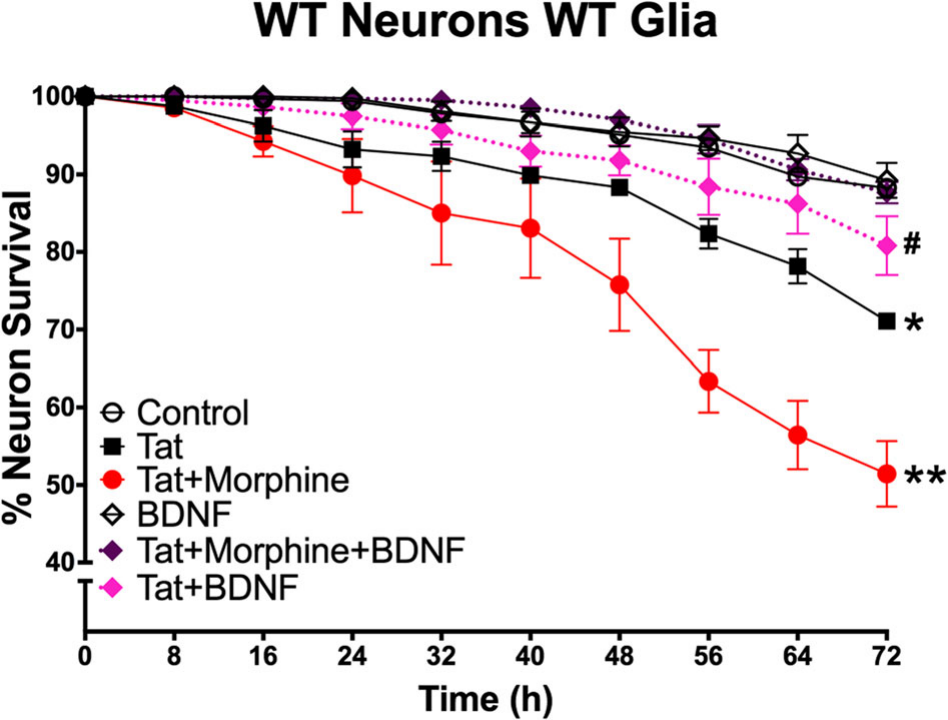
Exogenous BDNF protects against Tat + morphine treatment. Wild-type, mixed glial-neuronal co-cultures were treated with BDNF in conjunction with Tat or Tat + morphine co-treatment (represented by dotted survival curves). Tat alone was toxic compared to no treatment (*p < 0.05), and Tat + morphine co-treatment was significantly more toxic than Tat treatment alone (**p < 0.0001). BDNF applied for 72 h was entirely protective against Tat and morphine co-exposure, reducing toxicity to control levels (*n* = 4). BDNF was partially protective against Tat alone. Survival of neurons treated with Tat + BDNF was not significantly different from either controls or cultures treated with Tat alone (#) (*n* = 4)

BDNF was applied to co-cultures to see if it would promote the survival of striatal neurons co-exposed to Tat + morphine. Co-cultures of wild-type neurons and glia were treated with BDNF concurrently with combined Tat and morphine for 72 h. Time-lapse analysis demonstrated that exogenous BDNF was partially protective against the neurotoxic effects of Tat alone, which was not the case for CCR5 deficiency. However, similar to CCR5 deficiency, BDNF reversed the combined neurotoxic effects of Tat + morphine (Fig. 6). BDNF alone at this concentration did not increase the survival of neurons in untreated, wild-type cultures; survival of both was over 90%.

### Loss of glial CCR5 expression produces a shift in proBDNF/BDNF levels

**Fig. 7:**
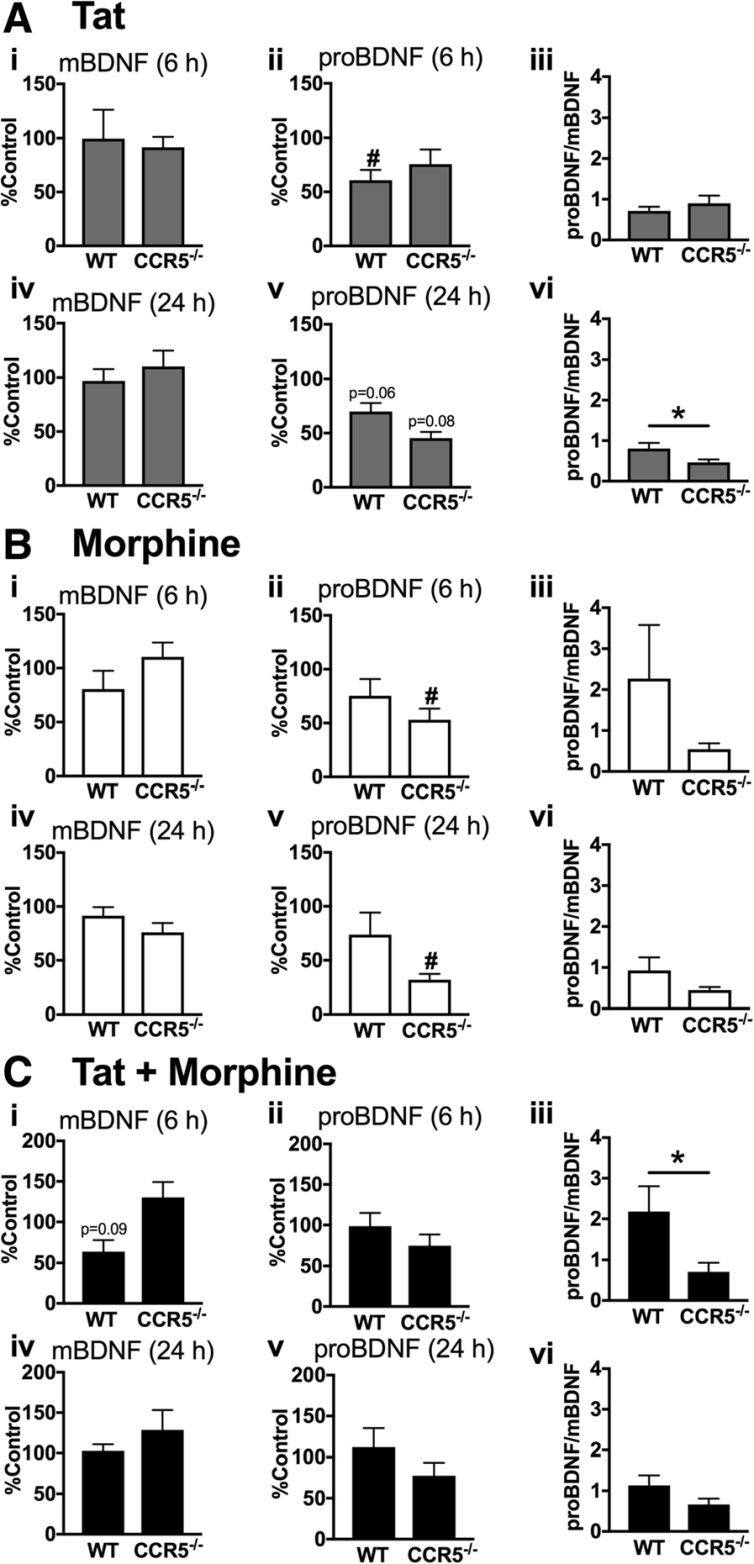
Loss of glial CCR5 expression produces a shift in proBDNF/mBDNF levels. mBDNF and proBDNF levels were analyzed in conditioned media from wild-type or CCR5-deficient glia treated with Tat and/or morphine after 6 and 24 h to determine if the levels of proBDNF and mBDNF and their ratios were altered (*n* = 7–8). **a** Tat significantly reduced proBDNF in wild-type cultures compared to untreated control levels at 6 h (ii) with similar trends in both genotypes at 24 h (v). The ratio of proBDNF/mBDNF was significantly higher in wild-type cells at 24 h, suggesting reduced neuronal support (vi). **b** Morphine significantly decreased proBDNF in CCR5-deficient glia at both 6 and 24 h compared to control levels (ii, v). These decreases in proBDNF did not significantly alter the proBDNF/mBDNF ratios (iii, vi). **c** Tat + morphine treatment did not significantly alter mBDNF and proBDNF levels in glia of either genotype, although there was a strong trend to decrease mBDNF in wild-type cultures at 6 h (i). Nevertheless the treatment very significantly decreased the proBDNF/mBDNF ratio at 6 h (iii), suggesting enhanced protection with CCR5 deficiency. A slight trend towards this shift continued at 24 h (vi; *p* = 0.17). #*p* < 0.05 vs control; *p < 0.05 for all figures

Based on our prior studies demonstrating significant but reversible reduction in glial production of mBDNF after exposure to HIV-infected supernatant ± morphine [48], as well as other studies where HIV-1 gp120 altered BDNF processing [47], we analyzed levels of both mBDNF and its precursor, proBDNF, which binds p75NTR to activate cell death pathways. We also compared changes in their ratios. Wild-type and CCR5-deficient glial cultures were treated with Tat (Fig. 7a), morphine (Fig. 7b), or concurrent Tat and morphine (Fig. 7c) and harvested at 6- and 24-h time points for protein analysis. After Tat treatment, mBDNF levels measured by ELISA were unchanged from levels in media in untreated control cultures at both 6 h and 24 h (Fig. 7a (i, iv)). Tat by itself significantly reduced proBDNF in wild-type cultures versus control cultures at 6 h, with a strong trend towards reduction in both wild-type and CCR5-deficient cultures at 24 h (Fig. 7a (ii, v)). Morphine by itself reduced only proBDNF and only in CCR5-deficient cultures (Fig. 7b (ii, v)). The combination of Tat and morphine showed a strong trend to reduce mBDNF in wild-type cultures at 6 h (Fig. 7c (i)) and was the only treatment to affect mBDNF. The ratio of proBDNF to mBDNF has been used as one index of relative neurotrophic support [47]. CCR5 deficiency strongly reduced this ratio by over twofold at 6 h in cells treated with Tat and morphine (Fig. 7c (iii)), and the protection of neurons in the CCR5-deficient glial environment may reflect the relative increase in mBDNF. A similar trend noted at 24 h was noted (*p* = 0.17) (Fig. 7c (vi)). The only other significant change in this ratio was a much smaller, but still significant, decrease in CCR5-deficient cultures treated with Tat (Fig. 7a (vi)).

## Discussion

Mild-to-moderate HAND still occurs in roughly 50% of HIV-infected individuals who receive typical antiretroviral therapy, in part due to relatively low penetration of cART drugs through the blood-brain barrier but also due to the extended lifespan afforded by long-term cART treatment. Even in patients where viral replication is undetectable, the release of toxins such as HIV-1 Tat, an early viral protein required for the transactivation of HIV transcription, can persist. While Tat may have direct excitotoxic effects on neurons, particularly well-documented in purified neuronal cultures [30, 49], it also fosters an environment of chronic inflammation through effects on astroglia and microglia. This may result in indirect neurotoxicity through the production of reactive oxygen and nitrosative species and via the release of proinflammatory chemokines and cytokines [50–52], triggering a cascade of inflammatory events that is ultimately damaging to neurons. Furthermore, Tat is present in measurable levels circulating in the blood and CSF of HIV patients [53, 54], suggesting that it is a clinically relevant surrogate for some aspects of HIV neuropathogenesis, especially in virally suppressed patients.

CCR5 is a chemokine receptor that also functions as a major co-receptor for HIV entry. It is normally expressed at low levels by glia throughout the CNS, but this expression can be upregulated by HIV-1 and by Tat [55]. CCR5 expression can also be upregulated in an additive or interactive manner with co-exposure to Tat and the preferential MOR agonist morphine [56]. We have been interested in understanding the role of the CCL5-CCR5 axis in mediating HIV and Tat-induced inflammation and neurotoxicity, with a special interest in whether moderating this chemokine system might be useful in reducing neurotoxic interactions between HIV-1 Tat and opiate exposure. We showed that intrastriatal delivery of Tat caused a local inflammation, characterized by astroglial expression of CCL2 (MCP-1) and 3-nitrotyrosine expression in microglia that was exacerbated by co-administering time-release, subcutaneous morphine pellet implants. Microglial 3-nitrotyrosine and astroglial expression of CCL2 were not seen in a CCL5 (RANTES)-deficient mouse, strongly implicating CCL5-to-CCR5 signaling in the amplification of astroglial CCL2 production and resultant macrophage/microglial activation and recruitment [45]. In a related study, we also showed that pre-treating Tat-inducible transgenic mice with maraviroc attenuated the withdrawal-mediated increase in levels of many of the cytokines that occurred in mice co-exposed to Tat and morphine and restored antinociceptive properties of morphine that were attenuated by Tat [57]. CCR5 loss is also protective against other HIV proteins, including gp120 [58], as assessed by reduced neuronal damage and microglial activation.

The present studies used both genetic (glia and neurons derived from a constitutive, CCR5-deficient mouse) and pharmacologic (CCR5 blocker maraviroc) approaches to demonstrate that CCR5 is directly involved in HIV neuropathogenic mechanisms irrespective of viral titers. That CCR5 plays a role in the inflammatory processes associated with many disease states, such as cerebral ischemia and reperfusion injury, and neuropathic pain is well-established [59, 60]. In HIV, the role of CCR5 in neurologic outcomes is more difficult to assign since CCR5 is a major co-factor for the infection process. A naturally occurring, 32 base-pair deletion in the CCR5 gene produces a nonfunctional protein, effectively rendering a knockout of the receptor. HIV+ individuals who possess one copy of this mutation show slower progression to AIDS and a reduced occurrence of HAND than those without the mutation [36, 37]. Studies in which maraviroc supplemented the antiretroviral therapy regimen in virally suppressed patients led to improvements in cognition [41, 61]. However, due to the role of CCR5 as a co-receptor for HIV entry, it is unclear if the reduced prevalence of HAND in this population is due to decreased inflammation or decreased progression of infection. In this study, we isolated the inflammatory effects of HIV-1 Tat without the confounding effects of viral infection and replication in monocytes and microglia. Since our paradigm is non-infectious, the findings establish that blocking CCR5 activity can significantly improve neuronal outcomes by mechanisms not involving reduced HIV infection.

As we hypothesized, when CCR5 was deleted from glia in mixed glial cultures, co-cultured neurons were protected against the enhanced degree of toxicity resulting from co-exposure to Tat and morphine. Surprisingly, when CCR5 was absent from glia, morphine appeared to entirely protect co-cultured neurons from the effects of Tat (Fig. 3b). These findings were further explored and confirmed in an alternative, pharmacological approach utilizing maraviroc to control the length of CCR5 blockade. When maraviroc was given to more mature co-cultures concurrent with Tat ± morphine treatments for 72 h, protection was seen against the interactive effects of Tat and morphine co-exposure. In co-cultures incubated with maraviroc for their entire developmental period in vitro, to mimic a constitutive knockout, neurons were additionally protected from Tat-induced toxicity when morphine was present (Fig. 4). Maraviroc may thus afford additional neuroprotection to individuals exposed to opioids, depending on timing and duration of maraviroc therapy. Notably, maraviroc blocked the increase in neuronal [Ca^2+^]_i_ seen at acute time points after exposure to Tat ± morphine (Fig. 5), suggesting that early events leading to neuronal dysfunction may be CCR5-dependent and reversible. Naloxone pretreatment revealed that both the predicted and unexpected protective effects of morphine were mediated by opioid receptors.

We explored a potential mechanism for the aforementioned protective effects against both Tat and Tat + morphine. BDNF, a neurotrophin that modulates development and survival of young neurons and is important in establishing and maintaining normal synaptic connectivity, has surfaced as a potential therapeutic target in HAND. Both microglia and astrocytes can be a major source of released BDNF [62, 63]. The mature form of BDNF (mBDNF) preferentially binds TrkB, through which it initiates PI3K and MAPK signaling pathways in neurons, as well as NF-κB-mediated activation of transcription factors [64, 65]. proBDNF binds the alternative p75NTR to initiate pro-apoptotic cascades [66]. Levels of mBDNF are reportedly reduced in HIV+ individuals and also modulated by drugs of abuse [47, 67, 68]. For example, morphine exposures in vivo appear to increase the extracellular protease tissue plasminogen activator, thus increasing cleavage of proBDNF to mBDNF. Withdrawal from morphine had the opposite effect [69]. Intracellular conversion of proBDNF to mBDNF in neurons is reduced by the HIV surface envelope protein gp120, which reduces levels of the intracellular protease furin [47]. Exogenous mBDNF can rescue neurons exposed to gp120 both in vivo and in vitro [70, 71], likely involving downregulation of CXCR4. In a prior study involving glia exposed to HIV-infective supernatant ± morphine, mBDNF levels, but not glial-derived neurotrophic factor (GDNF) levels, were significantly decreased. This reduction was reversed upon removal of HIV treatment [48]. The relatively higher proBDNF/mBDNF ratio measured in the medium of wild-type versus CCR5-null glial cultures exposed to Tat and morphine (Fig. 7c (iii)) is in line with the outcomes above since neurons co-cultured with CCR5-null glia were protected. We measured significant differences in proBDNF/mBDNF ratios at 6 h with trends at 24 h. These time points reflect prior studies that have demonstrated transient increases in BDNF mRNA and protein levels. For instance, response to cortical injury in rats involves an increase in BDNF mRNA from 1 h that begins to decline after 24 h [72, 73]. gp120 also leads to early changes in BDNF and proBDNF release from cerebellar or cortical neurons, from 1 h post-treatment [47]. As demonstrated by the persistently high percentage of neuronal survival in BDNF-treated co-cultures (Fig. 4), these rapid, transient neuroprotective signals may result in long-lasting effects even in the face of a complex milieu of secondary and tertiary factors by 72 h of treatment exposure. Activation of p75NTR, for instance, in multiple cell types has elucidated a role in immune response regulation. Injury to retinal ganglion neurons leads to the upregulation of p75NTR of nearby Müller glial cells, which can activate downstream cytokine production that is thought to eventually lead to the damage and demise of bystander neurons [74]. Our findings are further supported in other models of neuronal injury such as cerebral ischemia, in which the loss of CCR5 reduces long-term inflammatory injury, potentially through increases in BDNF levels [60, 75]. However, while we demonstrated that Tat and/or morphine treatment led to specific changes in mBDNF and proBDNF levels, shifts in p75NTR and TrkB levels may add a degree of complexity in determining the fate of the neurons, as receptor expression is responsive to injury in a time- and cell-dependent manner [76–78].

Opiates and HIV have historically been interlinked epidemics, and injection drug use carries an increased risk of contracting HIV. Moreover, opiates are used to manage HIV-related pain syndromes, which may impact HAND symptoms in virally suppressed individuals. Cooperative effects between HIV and opiates that increase CNS inflammation are well-documented, and many involve the HIV co-receptor CCR5. For example, opiates modulate CCR5 expression in a number of CNS and immune cells, including microglia [56], astrocytes [79], and peripheral monocytes [43], and can thereby increase rates of HIV infection as well as pro-inflammatory products and immune cell recruitment. The analgesic properties of MOR activation are also reduced when there is an abundance of CCR5 ligands [80]. MOR function is also altered by Tat exposure, which lowers morphine efficacy through decreased G-protein activation [81]. Reduced MOR signaling would tend to increase the amounts of opiates used by HIV-infected individuals for effective analgesia or for illicit effects.

In earlier studies, wild-type neurons co-cultured with MOR-deficient glia were completely protected against the exaggerated neurotoxic effects of co-exposure to Tat + morphine. These result were consistent with findings that the selective MOR antagonist β-funaltrexamine prevented morphine from exacerbating Tat-induced increases in proinflammatory cytokine transcripts in astroglia [27]. Surprisingly, morphine also afforded neuroprotection against Tat [30]. Given that the present results show strikingly similar protective effects in the absence of glial CCR5 (Fig. 3b), a possible interaction between CCR5 and MOR signaling that modulates neuroprotection seems likely. Some interactions between opiates and CCR5 are likely to involve the heterologous interactions that can occur between G-protein-coupled receptors (GPCRs) and that have been shown for MOR and CCR5 [82, 83]. Heterologous desensitization may explain a reduced chemotactic effect of CCR5 by pretreatment with MOR agonists [80]. Recently, synthetic, bivalent ligands composed of maraviroc and naltrexone that target the proposed CCR5-MOR heteromers/oligomers have shown that such interactions may occur in a cell type-specific manner [84–86]. The inability to form MOR-CCR5 heterodimers in CCR5-null (or MOR-null) glia may alter the cellular response to concurrent morphine exposure, contributing to neuroprotection. Although such a response might additionally involve contributions from κ-(KOR) or δ-(DOR) opioid receptors, morphine has lower affinity at KOR or DOR (than MOR). Prior studies also indicate that MOR is largely responsible for the morphine and Tat-induced inflammation and bystander neurotoxicity that is mediated by glia [28, 30].

This study establishes a role of glial CCR5, unrelated to infective processes, in mediating neurotoxicity due to HIV-1 Tat and the interactive effects of Tat and morphine. The differential toxic versus protective effects of morphine in the presence or absence of CCR5 hint at complex relationships that may involve heterologous interactions between CCR5 and MOR. Other results infer a role for BDNF, and perhaps an altered balance between proBDNF and mBDNF levels, in some aspects of the protection. These studies are particularly pertinent to HIV+ individuals who are virally suppressed, yet still develop mild neurocognitive deficits via ongoing, low levels of neuroinflammation in the CNS that involve CCR5 activation.

## Conclusion

HIV antiretroviral therapy has been successful in limiting AIDS-related complications, but neurocognitive deficits due to the inflammation driven by early CNS viral penetration persist in up to 50% of HIV-infected individuals. Opiate exposure, which is common in the HIV+ population, worsens the severity of HAND. The cellular mechanism(s) that exacerbate inflammation and CNS disease remain largely unexplained. We tested the hypothesis that the CCL5-CCR5 axis plays a pivotal role in opiate exacerbation of HIV deficits using both genetic and pharmacological approaches. Loss of glial, but not neuronal, CCR5 protected striatal neurons from HIV-1 Tat and morphine co-exposure. Importantly, loss of CCR5 not only blocked the interactive effects of Tat and morphine, but also reversed the Tat toxicity seen in the cultures as long as morphine was present. These findings were confirmed with both long- and short-term exposure to the CCR5 antagonist maraviroc. We measured a shift in the ratio of proBDNF/mBDNF released from CCR5-deficient glia that favored neuroprotection, and exogenous BDNF protected neurons from HIV-1 Tat and morphine exposure in a manner that mirrored the effects of CCR5 deficiency. Our findings suggest that CCR5 is involved in processes that impact neuronal survival and function unrelated to its important role in HIV infection, at least partly by altering the balance between proBDNF and mBDNF levels. The differential toxicity of morphine/opioids in the presence or absence of CCR5 also hints at complex relationships between CCR5 and MOR that may involve heterologous interactions. Our findings are pertinent in terms of understanding and treating HIV+ individuals who develop neurocognitive deficits even though their peripheral viremia is well-controlled.

## Abbreviations

[Ca^2+^]_i_: Intracellular calcium concentration
ANOVA: Analysis of variance
BDNF: Brain-derived neurotrophic factor
cART: Combined antiretroviral therapy
CCL2: C-C chemokine ligand 2
CCR5: C-C chemokine receptor 5
CNS: Central nervous system
DOR: δ-opioid receptor
ELISA: Enzyme-linked immunosorbent assay
GDNF: Glial-derived neurotrophic factor
Gp120: Glycoprotein 120
GPCR: G-protein-coupled receptor
HAND: HIV-associated neurocognitive disorders
HIV: Human immunodeficiency virus
KOR: κ-opioid receptor
MAPK: Mitogen-activated protein kinase
MOR: μ-opioid receptor
NF-κB: Nuclear factor kappa-light-chain-enhancer of activated B cells
NTR: Neurotrophin receptor
PI3K: Phosphoinositide 3-kinase
ROI: Region of interest
Tat: HIV-1 transactivator of transcription
tPA: Tissue plasminogen activator
TrkB: Tyrosine receptor kinase B
